# Nuclear receptor 4A1 is critical for neutrophil-dependent pulmonary immunity to *Klebsiella pneumoniae* infection

**DOI:** 10.3389/fimmu.2025.1558252

**Published:** 2025-03-06

**Authors:** Norie Sugitani, Matthew Henkel, Jessica Partyka, Alexander Applegate, Felicia Kemp, Craig A. Byersdorfer, Taylor Eddens, Brian T. Campfield

**Affiliations:** ^1^ Division of Pediatric Infectious Diseases, Department of Pediatrics, University of Pittsburgh School of Medicine, Pittsburgh, PA, United States; ^2^ Department of Pediatrics, Division of Blood and Marrow Transplantation and Cellular Therapies, University of Pittsburgh School of Medicine, Pittsburgh, PA, United States; ^3^ Department of Pediatrics, Division of Blood and Marrow Transplantation & Cellular Therapy, University of Minnesota Medical School, Minneapolis, MN, United States; ^4^ Division of Allergy and Immunology, Department of Pediatrics, University of Pittsburgh School of Medicine, Pittsburgh, PA, United States; ^5^ Department of Pediatrics, University of Pittsburgh Medical Center (UPMC) Children’s Hospital of Pittsburgh, Pittsburgh, PA, United States

**Keywords:** *Nr4a1*, Nur77, neutrophil, bacterial pneumonia, lung immunity, *Klebsiella pneumoniae*

## Abstract

**Introduction:**

Bacterial pneumonia is a burdensome, costly disease and increasingly challenging to treat due to antibiotic resistance. Complex host-pathogen interactions regulate protective immunity. Neutrophils play a central role in pulmonary bacterial immunity, and mechanistic understanding of neutrophil functions in bacterial pneumonia has potential clinical and fundamental application. Nuclear receptor 4a1 (Nr4a1), a member of the nuclear orphan receptor family, has been described to regulate inflammation and immune development in a cell type-specific manner, but its role in pulmonary host defense is not well understood.

**Methods:**

Wild-type (WT) and *Nr4a1^-/-^* mice, as well as bone marrow chimeric and Gr-1+ antibody depleted mice, were infected with Klebsiella pneumoniae and assessed for bacterial burden in the lung and spleen, gene transcription, protein levels, histology and cellular abundance by flow cytometry in the lung. WT and *Nr4a1^-/-^* neutrophils were exposed to live *Klebsiella pneumoniae* to quantify bacterial killing, as well as bulk RNA sequencing to assess transcriptomic differences.

**Results:**

Nr4a1-deficient mice are highly susceptible to Klebsiella pneumoniae pneumonia, which was mediated by *Nr4a1* expression in immune cells. Gr-1+ antibody depletion ameliorated the Nr4a1-dependent phenotype. *Ex vivo*, Nr4a1-deficient neutrophils had impaired bactericidal capacity, and transcriptomic analysis identified an Nr4a1-dependent host defense program in neutrophils.

**Discussion:**

Neutrophil *Nr4a1* expression is critical for defense against *K. pneumoniae* infection by regulating the neutrophil transcriptome. These findings suggest targeting *Nr4a1* signaling pathways in neutrophils may be useful for bacterial pneumonia treatment.

## Introduction

Pneumonia is the leading cause of death among children worldwide, responsible for 15% of overall child mortality, surpassed only by prematurity (1). In the US, pneumonia is the most common infectious cause of hospitalization and death, with annual costs in excess of $17 billion (2–5). While antibiotics are the standard treatment for bacterial pneumonia, antibiotic resistance (AR) (6–9) is increasingly common, and AR pathogen treatment is associated with increased financial costs, morbidity and mortality (10–12). *Klebsiella pneumoniae*, a common cause of hospital-acquired pneumonia, is a U.S. Centers for Disease Control and Prevention-defined “Urgent Threat” to public health due to its virulence and increasing rate of antibiotic resistance (12). Improved treatments for AR pneumonia are urgently needed. The pathophysiology of pneumonia involves complex host-pathogen interactions, and insight into the critical mediators of host immunity holds the promise for developing immune-targeting treatments as novel therapeutic strategies, approaches that have transformed the fields of cancer biology and autoimmunity.

Neutrophils and macrophages are key myeloid immune populations in pulmonary antibacterial immunity, but the cell-specific molecular mechanisms remain only partially understood (13, 14). Nuclear receptor 4a1 (Nr4a1 or Nur77) is a steroid thyroid receptor family member of the orphan nuclear receptor subfamily 4a. Nr4a1, Nr4a2 (Nurr1), and Nr4a3 (NOR-1), have been shown to regulate homeostatic myeloid cell development. Prior studies have shown that Nr4a1 modulates macrophage and T cell-mediated inflammation as well as modulating metabolic pathways in the context of autoimmunity and oncology to constrain proinflammatory signaling and promote tolerance via transcriptional and epigenetic regulation (15–26). Few studies have examined the role of Nr4a1 in pulmonary host defense, specifically in the context of common bacterial pathogens. Thus, we sought to investigate whether Nr4a1 was critical for pulmonary immunity to pneumonia using the murine model of *Klebsiella pneumoniae (* 15, 17, 22, 26–28). This work identified that *Nr4a1* deficiency reduced survival, increased lung bacterial burden, and enhanced bacterial dissemination. We further observed that neutrophil-specific *Nr4a1* is critical for defense against *K. pneumoniae* by regulating a defense response transcriptional program. These findings suggest that targeting neutrophil-specific Nr4a1 could offer a promising avenue for developing novel immunotherapies against bacterial pneumonia.

## Methods

### Animals

All animal protocols were approved by the University of Pittsburgh Institutional Animal Care and Use Committee (IACUC, protocols #22061470 and #23104015), and all experiments were conducted in accordance with the guidelines and regulations set forth in the Animal Welfare Act (AWA) and PHS Policy on Humane Care and Use of Laboratory Animals. WT C57BL/6J and *Nr4a1^-/-^* (B6;129S2-*Nr4a1^tm1Jmi^*/J) mice were purchased from the Jackson Laboratory. Age- and sex-matched male and female mice between 8-12 weeks old at the beginning of experiments were used for this study. Ages of BM donor mice ranged between 8-16 weeks old.

### *Klebsiella pneumoniae* infection/bacterial burden analysis

*In vivo* infection of *K. pneumoniae 396* (*Kp396*), a hypervirulent mucoid clinical isolate (29), via intratracheal injection and bacterial burden quantification in left lung lobe and whole spleen were performed as described previously (30) except that each animal received *Kp396* at 5,000 – 10,000 CFU.

### Histology

Lungs were fixed in 4% paraformaldehyde and embedded in paraffin and sectioned at 4 µm. Lung sections were stained with hematoxylin and eosin (H&E). H&E-stained slides were scored for bacterial burden in a blinded fashion with respect to neutrophil infiltration in alveoli, neutrophil infiltration in interstitial, hyaline membrane, alveolar wall thickening, and abscesses (**Supplementary Table S1**) (31).

### Quantitative PCR

Right upper lung lobes were processed for qPCR as described previously (30). See **Supplementary Table S2** for primer assay information.

### Flow cytometry

Right middle and lower lung lobes were processed and stained for flow cytometry as described previously (30). See **Supplementary Table S3** for antibody information.

### Enzyme-linked immunosorbent assay

Lung homogenates were subjected to anti-IL-1β (ThermoFisher Scientific 88-7013-22) and anti-MPO (R&D DY3667) by ELISA as described previously (30).

### Bone marrow chimera generation

WT and *Nr4a1^-/-^* mice were conditioned with 1100 cGy total body irradiation in a split dose of 550 cGy 3-4 hours apart from an X-ray source (X-rad 320; Precision X-ray, North Branford, CT), followed by intravenous infusion of 5x10^6^ WT or *Nr4a1^-/-^* bone marrow (BM) cells. Transplanted mice were housed in sterile microisolator cages. BM cells were allowed to engraft for 8 weeks prior to infection.

### Isolation of primary BM neutrophils and macrophages/*K. pneumoniae* killing assay

Primary neutrophils were isolated from WT and *Nr4a1^-/-^* BM using 40/70% Percoll gradient (Fisher) following red blood cell lysis with increasing NaCl concentrations (0.2% followed by 1.6%). For BM macrophage isolation, red blood cell lysed BM cells with ACK lysis buffer (Gibco) was cultured for 7 days at 37 °C in 5.0% CO_2_ and DMEM media supplemented with 10% fetal bovine albumin (FBS), 100 U/ml penicillin, 100 μg/ml streptomycin, 292 μg/ml L-glutamine (all Gibco), and 20 ng/mL M-CSF (Life Technologies). Isolated neutrophils and macrophages were incubated with *Kp396* at an MOI of 10 in the killing assay media (IMDM media supplemented with 10% FBS, 292 μg/ml L-glutamine, 1x MEM non-essential amino acids, 10 mM HEPES, and 1 mM sodium pyruvate (all Gibco)) for 1 hour in the 37 °C with 5.0% CO_2_ on an orbital shaker at 250 rpm. Resulting supernatant was subjected to bacterial burden analysis as described above. Remaining cells were stored for gene expression analysis.

### RNA sequencing

Primary neutrophils were isolated via negative magnetic selection from WT and *Nr4a1^-/-^* BM using EasySep™ Mouse Neutrophil Enrichment Kit (StemCell Technologies) following manufacturer’s instructions. Cells were incubated with or without *Kp396* at a MOI of 0.4 in the killing assay media (IMDM media supplemented with 10% FBS, 292 μg/ml L-glutamine, 1x MEM non-essential amino acids, 10 mM HEPES, and 1 mM sodium pyruvate (all Gibco)) for 30 minutes at 37 °C prior to RNA isolation using RNeasy Micro Kit (Qiagen).

RNA was assessed for quality using an Agilent TapeStation 4150/Fragment Analyzer 5300 and RNA concentration was quantified on a Qubit FLEX fluorometer. Libraries were generated with the Illumina Stranded mRNA Library Prep kit (Illumina: 20040534) according to the manufacturer’s instructions. Briefly, 50 ng of input RNA was used for each sample. Following adapter ligation, 15 cycles of indexing PCR were completed, Illumina RNA UD Indexes. Library quantification and assessment was done using a Qubit FLEX fluorometer and an Agilent TapeStation 4150/Fragment Analyzer 5300. Libraries were normalized and pooled to 2 nM by calculating the concentration based off the fragment size (base pairs) and the concentration (ng/μl) of the libraries.

Sequencing was performed on an Illumina NextSeq 2000, using a P2 flow cell. The pooled library was loaded at 750 pM and sequencing was carried out with read lengths of 58 bp, with a target of 30 million reads per sample. Sequencing data was demultiplexed by the on-board Illumina DRAGEN FASTQ Generation software (v3.10.12).

Raw reads underwent pre-alignment quality control (QC) and trimming. Reads were then aligned using STAR (2.7.8a) via Partek Flow Bioinformatics software. Transcript normalization and gene expression analysis were performed using DESeq2 with FDR step-up used for multiple test correction. Hierarchical clustering was performed in both supervised and unsupervised manners by genotype/treatment. Gene set enrichment analysis using gene ontogeny terms were generated by significant hits (FDR<0.001).

RNA-seq data has been deposited at the Gene Expression Omnibus (GEO) database (accession code: IN PROGRESS).

## Results

### Nr4a1-deficient mice are highly susceptible to *K. pneumoniae* pneumonia

**Figure 1:**
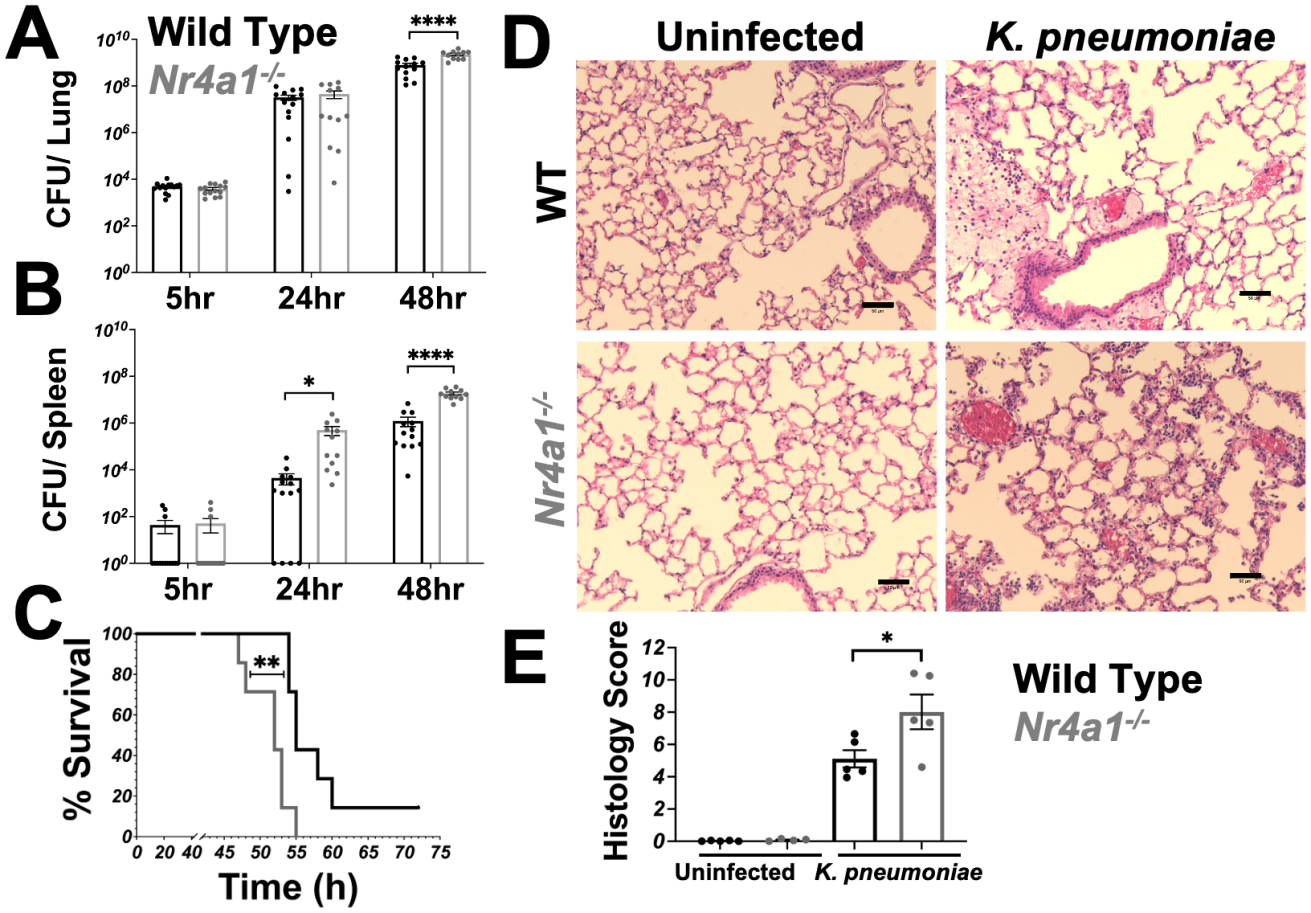
Nr4a1-deficient mice are highly susceptible to *K*. *pneumoniae* pneumonia. **(A, B)** WT and *Nr4a1^-/-^* mice were infected with *K*. *pneumoniae* intratracheally (IT). Colony forming unit (CFU) from lung **(A)** or spleen **(B)** homogenates were analyzed 5, 24, or 48 hours post infection. **(C)** Survival of WT and *Nr4a1^-/-^* mice following *K*. *pneumoniae* infection (n=7-8/group). **(D, E)** H&E staining of lungs 24 hours post infection ± *K*. *pneumoniae*. **(D)** Representative images of lung pathology. Scale bars indicate 50 μm. **(E)** Quantification of histology score. n= 11-15 **(A, B)**, 7-8 **(D)** 5 **(E)**, mean ± SEM., comparison by unpaired *t*-test **(A-B, E)** or Mentel-Cox test **(C)** where *p < 0.05, **p < 0.01 and ****p < 0.0001.

To evaluate the role of Nr4a1 in pulmonary infection, wild-type (WT) and *Nr4a1^-/-^* mice were infected with a clinical isolate of *Klebsiella pneumoniae (Kp396) (* 29). We found that *Nr4a1* transcription increased in WT mice following *K. pneumoniae* infection while *Nr4a1^-/-^* mice had no detectable Nr4a1 gene transcription (**Supplementary Figure S1A**). *Nr4a1^-/-^* mice exhibited a significantly increased bacterial burden in the lungs compared to WT mice (**Figure 1A**). Additionally, there was a significantly higher bacterial burden in the spleens of *Nr4a1^-/-^* mice (**Figure 1B**), indicating greater bacterial dissemination. This increased bacterial burden and dissemination was associated with a reduced survival rate in *Nr4a1^-/-^* mice (14% vs. 0%) at 72 hours post-infection (**Figure 1C**). Consistent with these findings, histological analysis identified greater infection-driven lung pathology in *Nr4a1^-/-^* mice (**Figures 1D, E**). These findings demonstrate the critical importance of *Nr4a1* in controlling *K. pneumoniae* pneumonia.

### Nr4a1 expression in neutrophils is protective against *K. pneumoniae* infection

As shown in **Supplementary Figure S1B**, transcripts of cytokines and chemokines essential for neutrophil activation and recruitment were reduced in the lungs of *Nr4a1^-/-^* mice. Further, prior to differences in lung or spleen bacterial burden, *Nr4a1^-/-^* mice had reduced neutrophil recruitment to the lungs, while the recruitment of alveolar and inflammatory macrophages was not different between WT and *Nr4a1^-/-^* mice following *K. pneumoniae* infection (**Supplementary Figure S1C**). Additionally, the expression of proteins associated with neutrophil effector functions (IL-1ß and MPO) were impaired in the lungs of *Nr4a1^-/-^* mice following *K. pneumoniae* infection (**Supplementary Figure S1D**). Together, these data demonstrate that *Nr4a1^-/-^* mice exhibit multiple aspects of impaired neutrophil-associated immune responses following *K. pneumoniae* infection.

**Figure 2:**
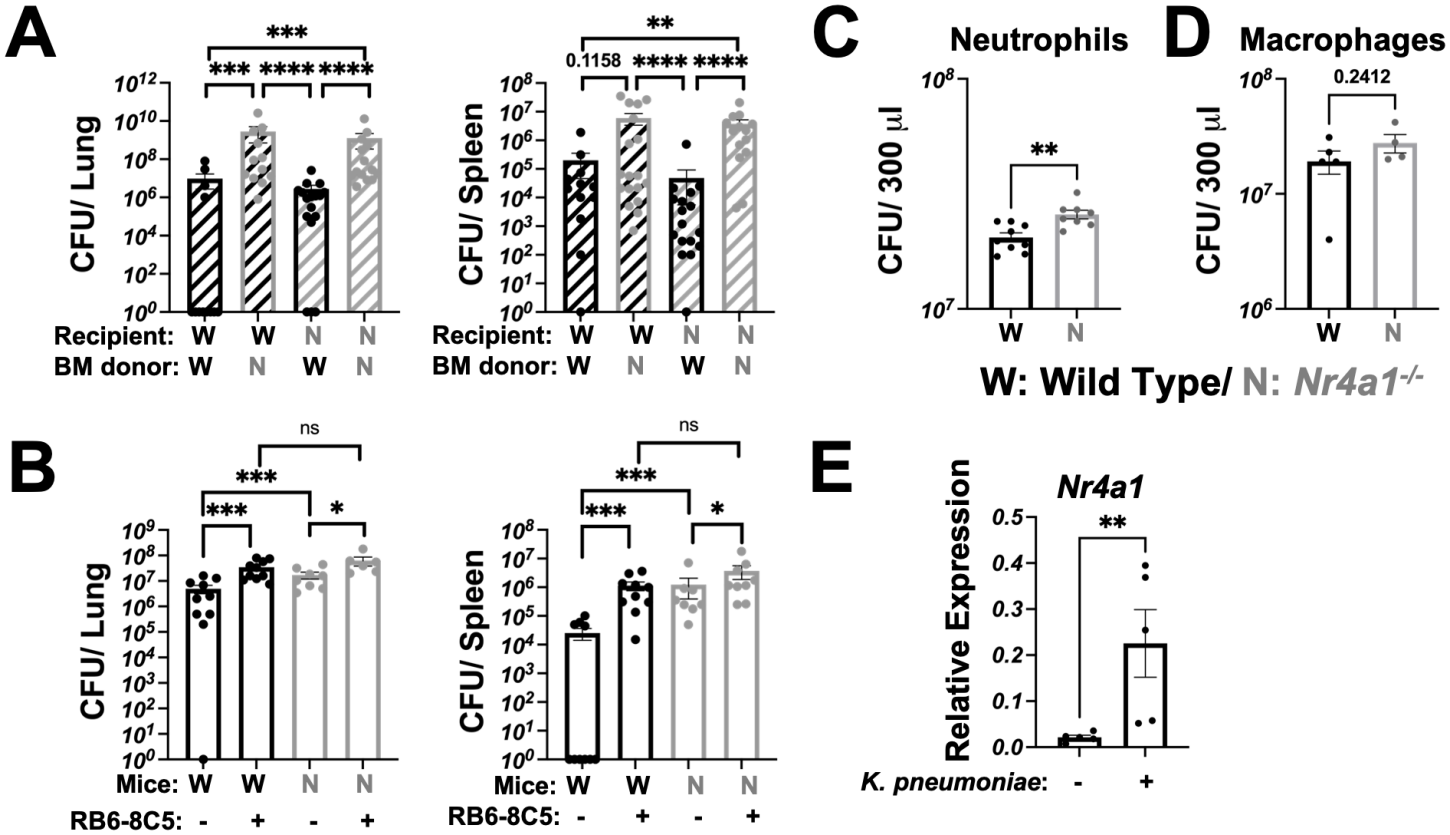
Nr4a1 expression in neutrophils is protective against *K*. *pneumoniae* infection. **(A)** Bone marrow (BM) chimera mice were generated using WT and *Nr4a1^-/-^* mice as either donor, recipient or both, as denoted. *K*. *pneumoniae*-infected burden was quantified by CFU in lung (left) or spleen (right) of recipient mice 48 hours post-infection. **(B)** WT and *Nr4a1^-/-^* mice were treated with 100 μg RB6-8C5 antibody (or isotype control) to deplete Gr-1^+^ cells. 24 hours post treatment, WT and *Nr4a1^-/-^* mice were infected with *K*. *pneumoniae* and bacterial burden in lung (left) or spleen (right) quantified 48 hours post infection. Purified BM primary neutrophils **(C)** or macrophages **(D)** from WT or *Nr4a1^-/-^* mice were incubated with *K*. *pneumoniae* at an MOI of 5 for 1 hour *ex vivo* at 37 °C, with supernatants were collected for *K*. *pneumoniae* quantification by CFU. **(E)** Expression of *Nr4a1* gene in purified WT neutrophils ± *K*. *pneumoniae* incubation for 1 hour at 37 °C via qPCR. n = 11-15 **(A)**, 6-10 **(B)**, 8-9 **(C)**, 4-5 **(D, E)**, mean ± SEM., comparison by Mann-Whitney tests (A/B) or unpaired *t*-test **(C-E)** where *p < 0.05, **p < 0.01, ***p < 0.001, and ****p < 0.0001.

To experimentally validate whether hematopoietic versus non-hematopoietic Nr4a1 expression was required for protection against *K. pneumoniae* pulmonary infection, we generated bone marrow (BM) chimeric mice, where the BM of WT or *Nr4a1^-/-^* mice was transplanted into lethally irradiated WT or *Nr4a1^-/-^* recipients (**Figure 2A**, **Supplementary Figures S3A, B**). Regardless of the recipient genotype, mice transplanted *Nr4a1^-/-^* BM failed to control lung bacterial burden and dissemination (**Figure 2A**), indicating that *Nr4a1* expression in BM-derived cells is crucial for protection against *K. pneumoniae* pulmonary infection.

To further investigate the immune cell specific *Nr4a1* expression requirement, we directly examined the impact of Gr-1^+^ cell-depletion on bacterial burdens in WT and *Nr4a1^-/-^* mice. RB6-8C5 monoclonal antibody treatment depleted cells (primarily neutrophils) and led to significantly increased bacterial burden in lung and increased bacterial dissemination post-infection in both WT and *Nr4a1^-/-^* mice, reinforcing the central role of neutrophils in *K. pneumoniae* clearance (**Figure 2B**). Notably, there was no difference in lung or spleen bacterial burden between WT and *Nr4a1^-/-^* mice after Gr-1^+^ depletion, suggesting that Nr4a1-dependent susceptibility is mediated by Gr-1^+^ cells.

To isolate the Nr4a1-mediated effect within the key myeloid cell populations, we employed an *ex vivo* primary cell bacterial killing assay. Here, we confirmed that purified BM neutrophils (**Figure 2C**), but not BM macrophages (**Figure 2D**), are directly responsible for the killing of *K. pneumoniae ex vivo* in a *Nr4a1*-dependent manner (**Figures 2C, D**, **Supplementary Figure S3C**). Correspondingly, WT neutrophils upregulated *Nr4a1* gene expression upon incubation with *K. pneumoniae ex vivo* (**Figure 2E**), consistent with observations *in vivo* (**Supplementary Figure S1A**).

### Nr4a1 controls neutrophil defense response genes

**Figure 3:**
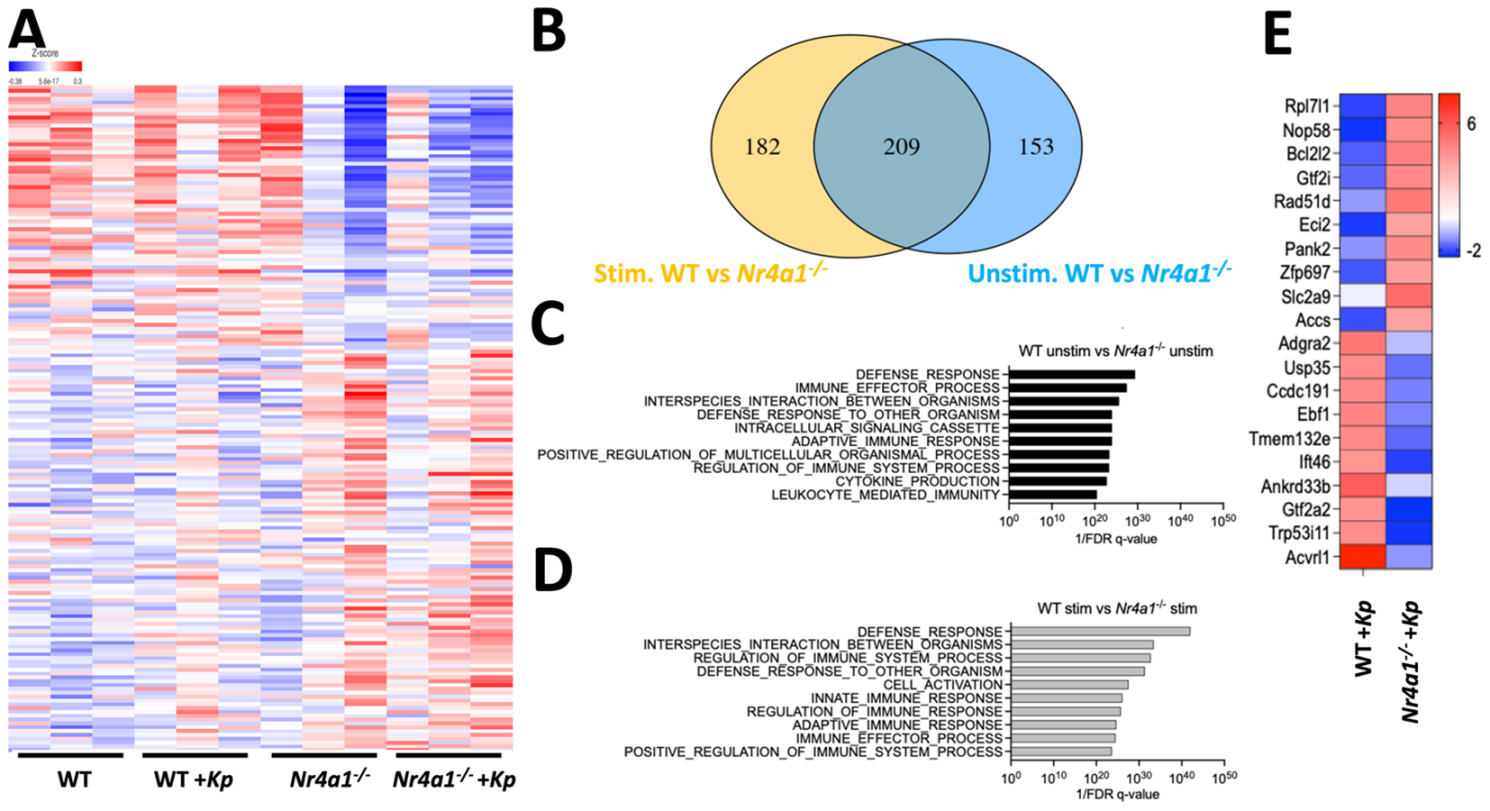
Nr4a1 regulates neutrophil defense response transcriptome. **(A-D)** RNA sequencing of WT and *Nr4a1^-/-^* primary neutrophils (PMNs). PMNs were isolated from bone marrow of indicated mice and incubated ± *K*. *pneumoniae ex vivo* for 30 minutes prior to RNA seq. **(A)** Heatmap comparing the transcriptome of *K*. *pneumoniae* stimulated and unstimulated WT and *Nr4a1^-/-^* PMNs. **(B)** Venn diagram of differentially expressed transcripts between *Kp-*stimulated (yellow) and unstimulated (blue) WT and *Nr4a1^-/-^* PMNs. GEO analysis of unstimulated **(C)** or stimulated **(D)** WT and *Nr4a1^-/-^* PMNs. **(E)** Heatmap of 20 most differentially expressed genes (fold change) comparing WT and *Nr4a1^-/-^ Kp-*stimulated neutrophils.

To investigate how neutrophil-specific *Nr4a1* expression contributes to defense against *K. pneumoniae*, bulk RNA-sequencing was performed on purified WT and *Nr4a1^-/-^* neutrophils, both with and without incubation with *K. pneumoniae ex vivo*, which revealed transcriptomic differences between WT and *Nr4a1^-/-^* PMNs, regardless of *K. pneumoniae* stimulation (**Figure 3A**, **Supplementary Figure S5**). 209 differentially expressed genes (DEGs) were identified in both unstimulated and *Kp*-stimulated conditions (DEGs) including *Nr4a1* as expected, as well as innate antibacterial effector genes *Tlr2*, *Myd88*, *Sod2*, *Ctss*, and *Fpr2* (**Figure 3B**; **Supplementary Table S5**). 182 DEGs were identified only under stimulated conditions, including phagocyte activation genes *Nod2*, *Elmo2*, *Rel* and *Ccl3* while 153 DEGs were identified under baseline conditions.

Pathway analysis of WT and *Nr4a1^-/-^* PMNs DEGs at baseline were enriched for terms associated with several neutrophil functions, including “Defense response,” “Immune effector process” and “Interspecies interaction between organisms” (**Figure 3C**). After *K. pneumoniae* infection, WT and *Nr4a1^-/-^* differentially expressed neutrophil transcripts yielded further enrichment for the terms “Defense response”, “Interspecies interaction between organisms”, “Regulation of Immune System Process”, “Defense Response to other organism”, as well as “Cell Activation” and “Innate Immune Response” (**Figure 3D**). Thus, RNA sequencing analysis identified that prior to infection *Nr4a1^-/-^* neutrophils have altered antibacterial transcriptomic profiles, and that *K. pneumoniae* infection activates a transcriptional program that fails to enrich in the context of Nr4a1 deficiency (**Figure 3B**, **Supplementary Table S4**). Examining the 20 most differentially expressed transcripts in the context of *K. pneumoniae* stimulation (**Figure 3E**) identified genes with no reported role in neutrophil antibacterial function, to the best of our knowledge, though 19 of these DEGs have been shown to be expressed in neutrophils. Cumulatively, these RNA sequencing data align with the *in vitro* and *in vivo* observations that Nr4a1 deficiency impairs neutrophil lung recruitment and bactericidal activity against *K. pneumoniae*.

## Discussion

Nr4a1 exhibits diverse cellular expression and pleotropic functions in mammalian biology, with its role in regulating immune cell transcription and cellular function being well described (15–20). Most of these studies focused on its role in macrophages and T cells (21–26). Despite documented expression in human and murine neutrophils, the function of Nr4a1 in neutrophils remain underappreciated (32, 33). Ballasteros et al. identified that neutrophil gene expression and function are influenced by the tissue environment, showing that Nr4a1 expression was induced in lung-specific neutrophils, which was acquired after arrival in the lung (34). This finding in particular suggests a significant role for Nr4a1 in neutrophil-mediated lung immunity.

Our work uncovered a novel neutrophil-specific requirement for Nr4a1 in pulmonary immunity against *Klebsiella pneumoniae*. Nr4a1 deficiency led to delayed neutrophil recruitment to the lung, downregulated genes involved in neutrophil activation and recruitment, and reduced expression of proteins critical for effector functions *in vivo*. RNA-seq analysis of purified neutrophils identified Nr4a1-dependent, antibacterial transcriptional signatures, and Nr4a1-deficient neutrophils exhibited impaired bacterial killing *in vitro*.

This neutrophil-specific role of Nr4a1 in promoting anti-bacterial activity is notably distinct from its effects on other immune cell populations. In macrophages, Nr4a1 deficiency increased TNFα production (22), along with other proinflammatory cytokines (23), exacerbating inflammation driven atherosclerosis (24). Similarly, Nr4a1 expression in T cells has been shown to suppress inflammation associated with autoimmunity (25) and cancer (26). This unique cell-specific function of Nr4a1 in neutrophils suggests that targeting neutrophil-specific Nr4a1 could be a promising approach developing immunotherapies against bacterial pneumonia.

A deeper understanding of how Nr4a1 influences neutrophil recruitment, activation, and effector functions compared to other immune cells is critical for optimizing Nr4a1-directed pneumonia treatment strategies. Several innate immune signaling pathways are activated during bacterial infection, including Toll-like receptors (e.g.CD14/TLR4) and Nod-like receptors (35–37). Previous studies have shown that Nr4a1 limits MAPK-mediated NF-κB p65 phosphorylation and downstream inflammation in alveolar macrophages (21, 22). The present data identified 544 Nr4a1-dependent DEGs, some of which are well-established neutrophil-associated mediators of bacterial immunity, such as the innate pathogen recognition receptors Toll-like 2 (*Tlr2*) and formyl peptide receptor 2 (*Fpr2*), and *Myd88*, *Sod2*, and *Rel* which regulate intracellular signaling and effector function. However, the most highly differentially expressed transcripts have no established role in neutrophil-mediated bacterial immunity, though some have been described to mediate neutrophil-dependent disease in non-infectious pathologies, such as enoyl-CoA delta isomerase 2 (*Eci2*) in colorectal cancer and solute carrier family 2 member 9 (*Slc2a9*) in hyperuricemic neutrophil inhibition (38, 39). Whether these pathways are modulated by Nr4a1 in neutrophil-dependent immunity to pulmonary infection remains unknown, and further examination could provide valuable insights.

In addition to investigating the differential roles of Nr4a1 in distinct immune cell populations, it is crucial to determine whether the Nr4a1-mediated immune response is pathogen-specific in pneumonia. In our study, Nr4a1 deficiency did not affect macrophage recruitment or change bone marrow-derived macrophage bactericidal capacity against *K. pneumoniae*. However, in a murine *E. coli* lung infection model, Cui et al. reported that Nr4a1 deficiency increased alveolar macrophage phagocytosis and enhanced bacterial clearance (40), alongside an increase in macrophage abundance in BAL fluid, despite a reduction in neutrophils. This discordance suggests that Nr4a1 not only functions in a cell-specific manner, but that pathogen-specific immune responses this may govern the lung phenotype. Investigating Nr4a1-regulated immune responses to different pneumonic pathogens, such as *S. pneumoniae*, influenza, and *Aspergillus*, will help delineate the critical mechanisms underlying these phenotypes of host-pathogen interaction during pulmonary infection.

Finally, it is crucial to validate findings from mouse models of neutrophil biology in human neutrophils, given the known differences in granule formation and transcriptional and proteomic profiles. Additionally, the tissue-specific role of neutrophil-specific Nr4a1 expression remains unexplored and could be investigated in other sites commonly infected by *K. pneumoniae*, including the liver, abdomen, and urinary tract.

In summary, we have demonstrated that neutrophil-specific Nr4a1 is essential for protective immunity against *K. pneumoniae* pneumonia. Further, our study identifies the neutrophil transcriptome regulated by Nr4a1. Continued research into the cell- and pathogen-specific regulation by Nr4a1, along with a deeper understanding of its downstream molecular pathways, may inform the development of new immunotherapies for bacterial infections.

## Data Availability

The data presented in the study are deposited in the NCBI GEO repository, accession number GSE290097.
