# Supplementary material for: Nuclear receptor 4A1 is critical for neutrophil-dependent pulmonary immunity to Klebsiella pneumoniae infection
Source: Front Immunol. 2025 Mar 6;16:1558252. doi: 10.3389/fimmu.2025.1558252 (PMC11922696; doi:10.3389/fimmu.2025.1558252)
Supplement: Supplementary file 1 [file DataSheet1.docx]

**Supplementary Tables**

|  | **Score per field** | | |
| --- | --- | --- | --- |
| **Parameter** | **0** | **1** | **2** |
| A. Neutrophils in the alveolar space | none | 1–5 | >5 |
| B. Neutrophils in the interstitial space | none | 1–5 | >5 |
| C. Hyaline membranes | none | 1 | >1 |
| D. Abscesses | none | 1 | >1 |
| E. Alveolar septal thickening | <2x | 2x–4x | >4x |

**Table S1: Histology scoring rubric.** Score = [(20 × A) + (14 × B) + (7 × C) + (7 × D) + (2 × E)]/(number of fields × 100).

| **Gene** | **Source** | **Identifier** |
| --- | --- | --- |
| Nr4a1 (mouse) | ThermoFisher Scientific | Mm01300401_m1 |
| Il1b (mouse) | ThermoFisher Scientific | Mm00434228_m1 |
| Tnfa (mouse) | ThermoFisher Scientific | Mm00443258_m1 |
| Cxcl1 (mouse) | ThermoFisher Scientific | Mm04207460_m1 |
| Cxcl5 (mouse) | ThermoFisher Scientific | Mm00436451_g1 |
| Lcn2 (mouse) | ThermoFisher Scientific | Mm01324410_m1 |
| Hprt (mouse) | ThermoFisher Scientific | Mm03024075_m1 |

**Table S2: Primer assays for qPCR**

| **Antibody** | **Source** | **Identifier** |
| --- | --- | --- |
| APC anti-mouse/ human CD11b | BioLegend | 101212 |
| FITC anti-mouse Ly6G | BioLegend | 127606 |
| FITC anti-mouse Gr-1 | BioLegend | 108406 |
| BV786 anti-mouse CD11c | BD | 563735 |
| PerCP-Cy5.5 anti-mouse CD45.1 | BD | 561869 |
| PE anti-mouse CD45.2 | eBioscience | 12-0454-82 |
| ant-mouse CD16/ CD32 | invitrogen | 14-0161-85 |
| **Live/dead dye** | **Source** | **Identifier** |
| Aqua Live Dead | ThermoFisher Scientific | L34966 |
| eFluor780 | invitrogen | 65-0865 |

**Table S3: Antibodies for FACS**

**Table S4: Complete transcript list from RNA-seq**

See ‘Table S4.xlsx’

**Table S5: Total Differentially Expressed Gene List from RNA-Seq**

See ‘Venn.xlsx”

**Supplementary Figures**


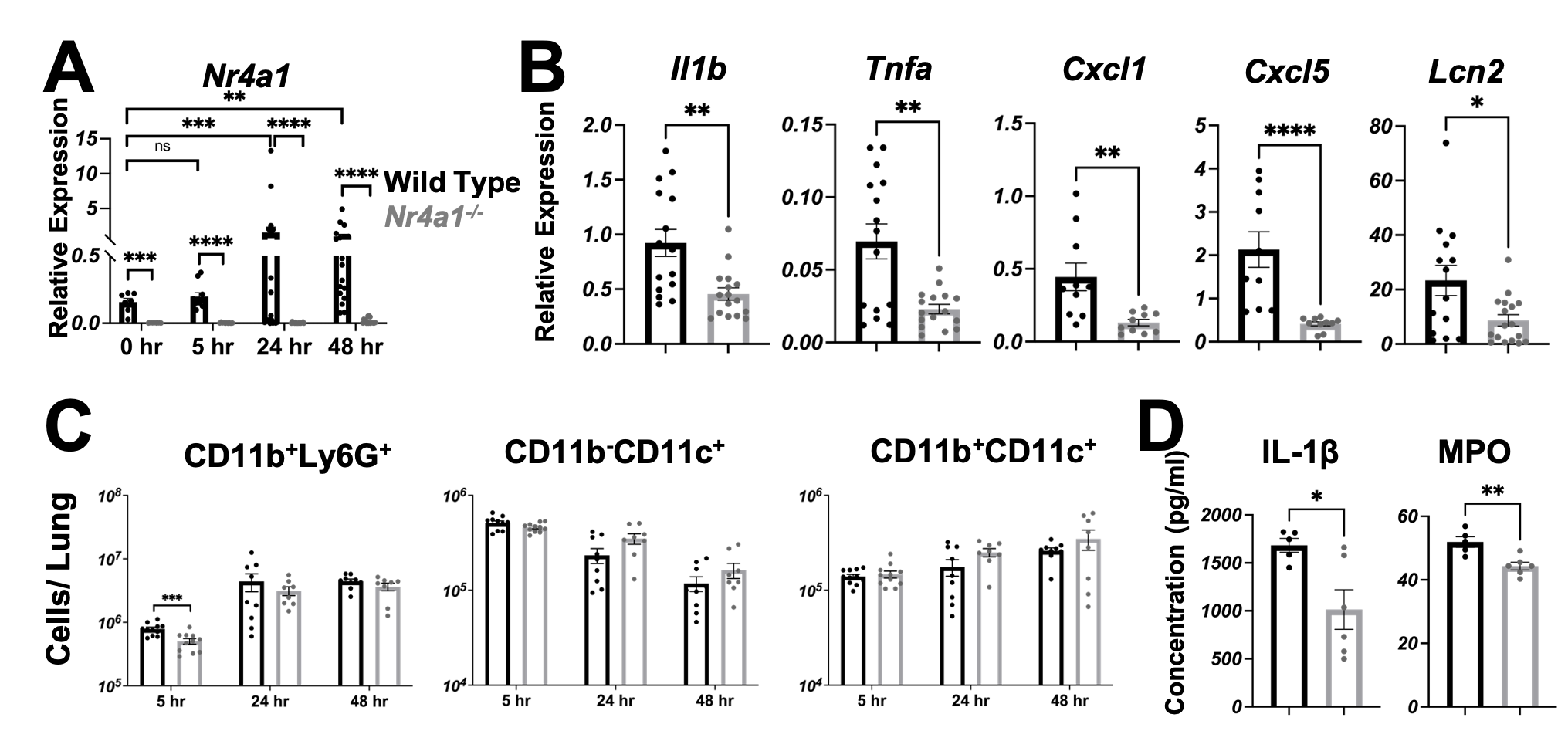


**Figure S1: Nr4a1^-/-^ mice have reduced pro-inflammatory cytokine and chemokine expression and delayed neutrophil recruitment to the lung.**

(A) Relative expression of *Nr4a1* gene in WT and *Nr4a1^-/-^* mice 0, 5, 24, or 48 hours post *K. pneumoniae* infection via qPCR. (B) Relative expression of indicated genes in WT and *Nr4a1^-/-^* mice 5 hours post *K. pneumoniae* infection via qPCR. (C) Population of CD11b^+^Ly6G^+^ (neutrophils), CD11b^-^CD11c^+^, and CD11b^+^CD11c^+^ cells in lungs of WT and *Nr4a1^-/-^* mice 5, 24, or 48 hours post *K. pneumoniae* infection as determined by FACS (see Figure S2 for gating strategy). (D) Concentrations of indicated proteins in WT and *Nr4a1^-/-^* mice 24 hours post *K. pneumoniae* infection via ELISA. n = 7-20 (A), 10-16 (B), 7-11 (C), 5-6 (D), mean ± SEM., comparison by Mann-Whitney tests (A-B, D) or unpaired T-tests (C) where*: p < 0.05, **: p < 0.01, ***: p < 0.001, and ****: p < 0.0001.

**
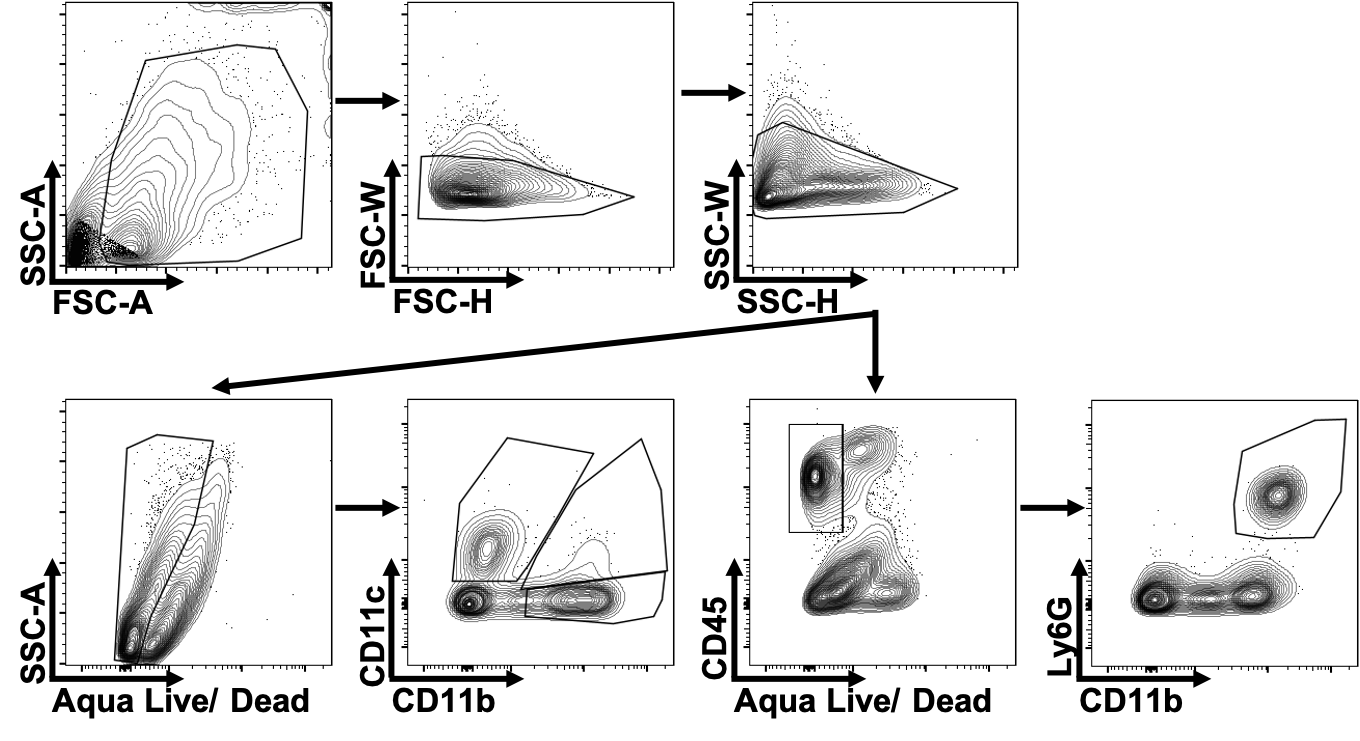
**

**Figure S2: FACS gating strategy – recruitment of immune cells to the lung.**

FACS gating strategy for Figure S1C.


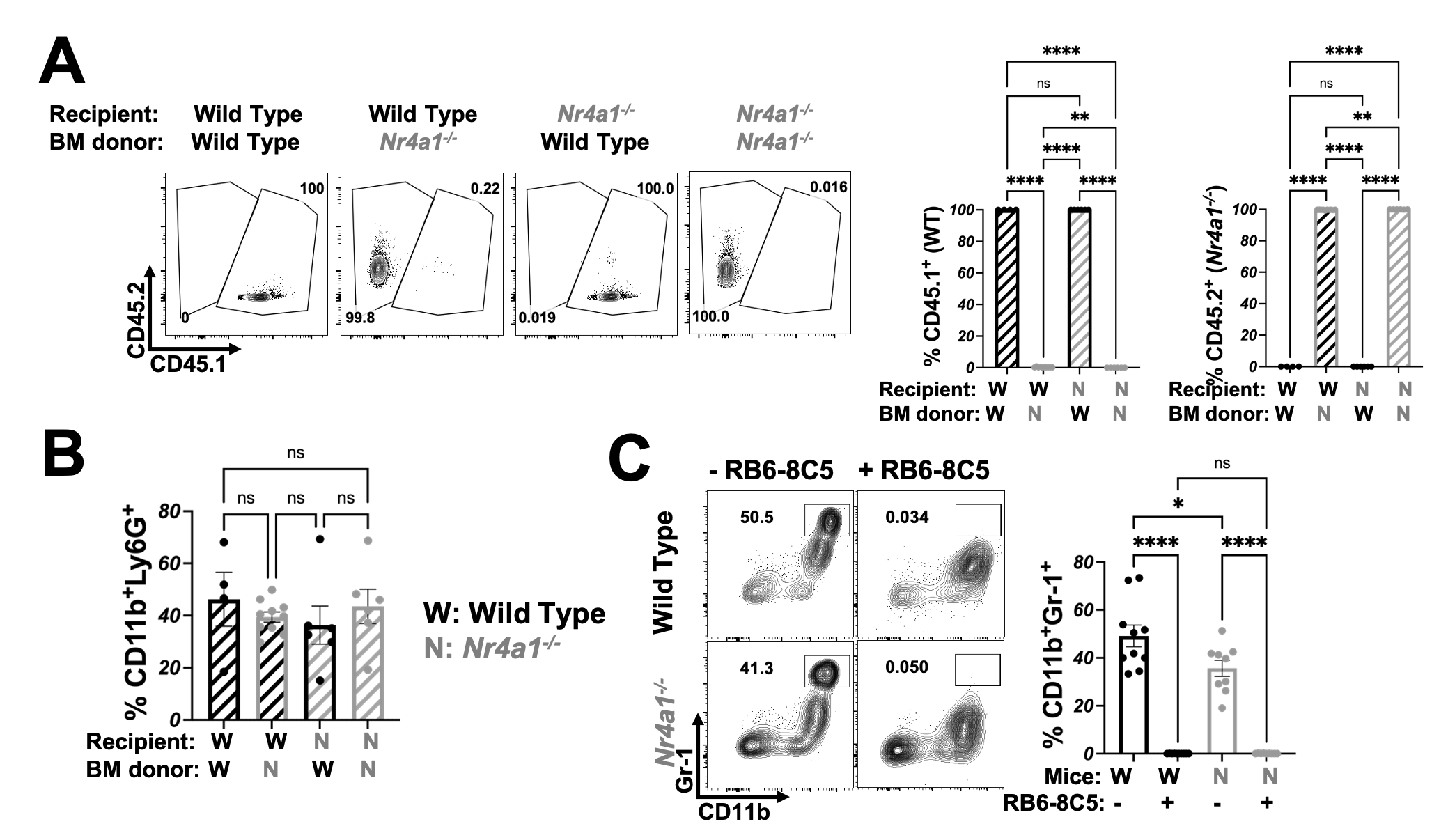


**Figure S3: Validation of *in vivo* pneumonia models.**

(A) % Chimerism of lung neutrophils in the BM chimera model described in Figure 2A. Right – representative CD45.2 (Nr4a1^-/-^) vs CD45.1 (WT) plots (see Figure S4 for gating strategy). Middle – quantification of WT BM derived neutrophils. Left – quantification of *Nr4a1^-/-^* BM derived neutrophils. (B) Recruitment of neutrophils (CD11b^+^Ly6G^+^) to lungs 48 hours post *K. pneumoniae* infection in the BM chimera model described in Figure 2A. (C) Validation of neutrophil depletion model described in Figure 2B. Right - representative Gr-1 vs CD11b plots (see Figure S4 for gating strategy) in lungs. Left – Quantification of neutrophils (CD11b^+^Gr-1^+^) in lungs 48 hours post *K. pneumoniae* infection. n = 4-10 (A/B), 10 (C), mean ± SEM., comparison by Tukey where*: p < 0.05, **: p < 0.01, and ****: p < 0.0001.


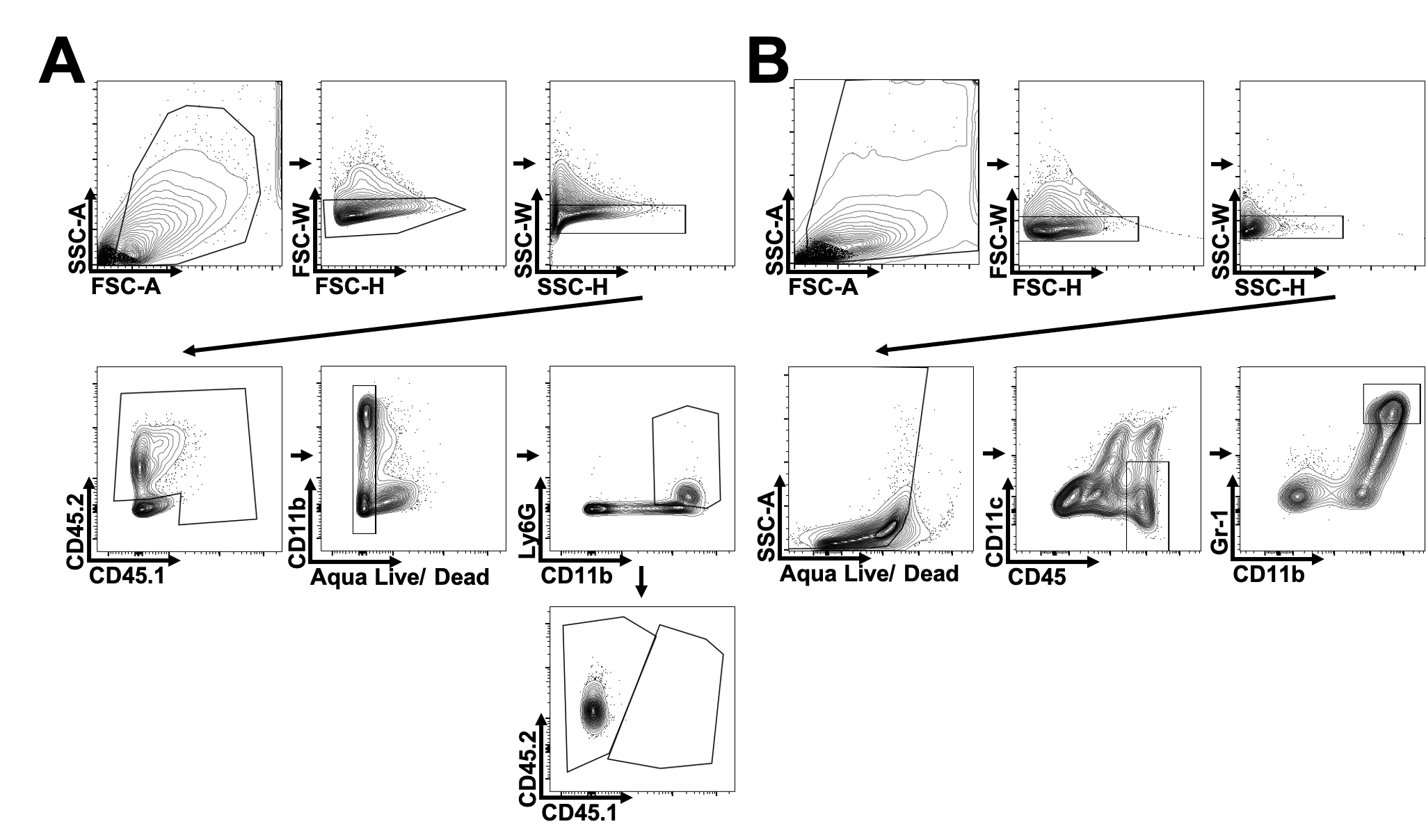


**Figure S4: FACS gating strategies.**

FACS gating strategies for (A) FigureS3A-B and (B) Figure S3C.

**
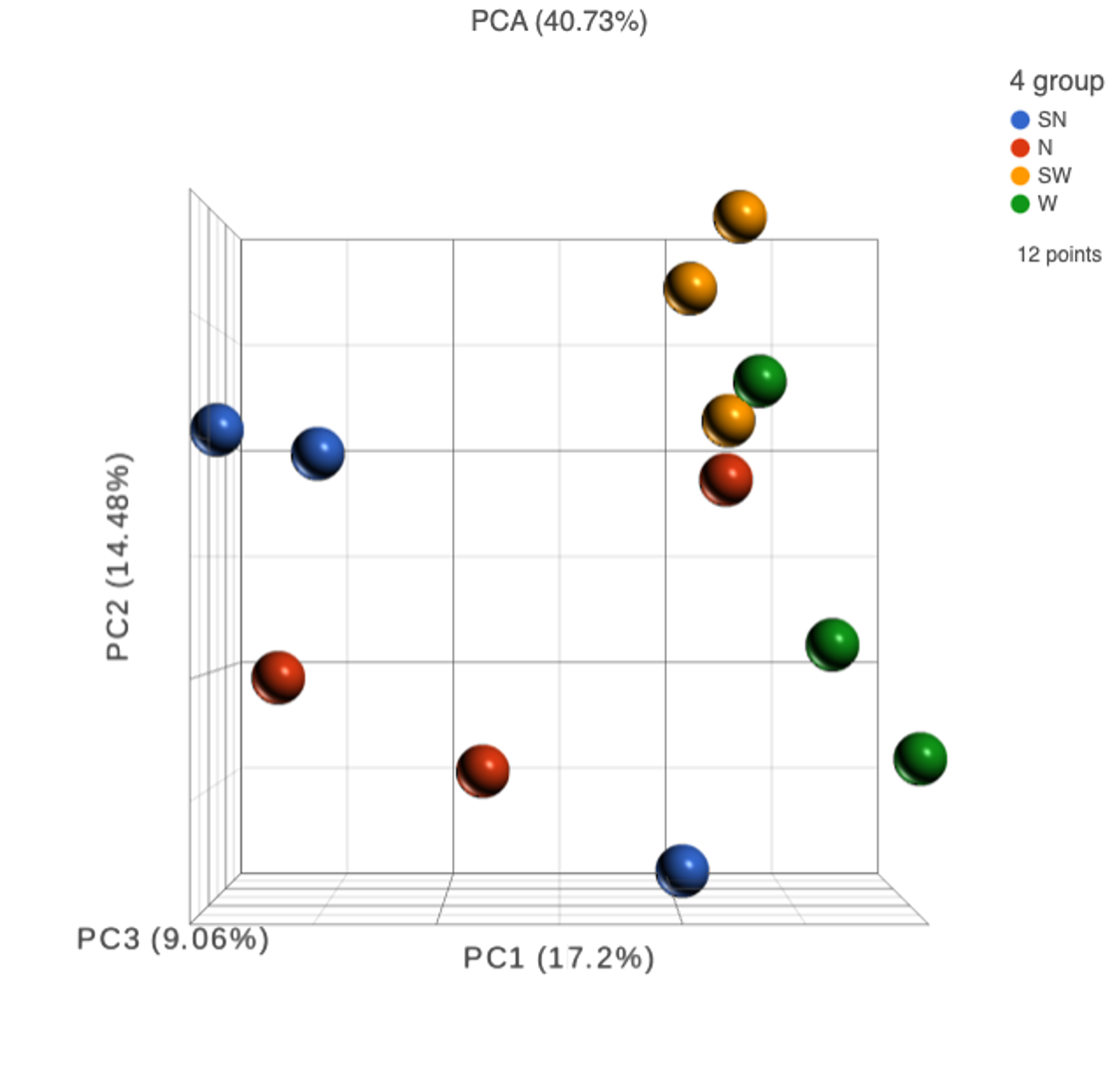
**

**Figure S5: PCA diagram from RNA-seq.**

Principle component analysis (PCA) of WT and *Nr4a1^-/-^* primary neutrophils (PMNs) from the RNA-seq data as described in Figure 3. SN= *K. pneumoniae* Stimulated Nr4a1^-/-^, N=unstimulated Nr4a1^-/-^, SW= *K. pneumoniae* Stimulated Wild Type, W= unstimulated Wild Type.
